# Green fluorescent protein as a scaffold for high efficiency production of functional bacteriotoxic proteins in *Escherichia coli*

**DOI:** 10.1038/srep20661

**Published:** 2016-02-11

**Authors:** Nagasundarapandian Soundrarajan, Hye-sun Cho, Byeongyong Ahn, Minkyung Choi, Le Minh Thong, Hojun Choi, Se-Yeoun Cha, Jin-Hoi Kim, Choi-Kyu Park, Kunho Seo, Chankyu Park

**Affiliations:** 1 Department of Animal Biotechnology, Konkuk University, Seoul 143-701, Korea; 2 Department of Infectious and Avian Diseases, Chonbuk National University, Iksan 570-752, Korea; 3 College of Veterinary Medicine, Kyungpook National University, Daegu 702-701, Korea; 4 College of Veterinary Medicine, Konkuk University, Seoul Seoul 143-701, Korea

## Abstract

The availability of simple, robust, and cost-effective methods for the large-scale production of bacteriotoxic peptides such as antimicrobial peptides (AMPs) is essential for basic and pharmaceutical research. However, the production of bacteriotoxic proteins has been difficult due to a high degree of toxicity in bacteria and proteolytic degradation. In this study, we inserted AMPs into the Green fluorescent protein (GFP) in a loop region and expressed them as insoluble proteins in high yield, circumventing the inherent toxicity of AMP production in *Escherichia coli*. The AMPs inserted were released by cyanogen bromide and purified by chromatography. We showed that highly potent AMPs such as Protegrin-1, PMAP-36, Buforin-2, and Bactridin-1 are produced in high yields and produced AMPs showed similar activities compared to chemically synthesized AMPs. We increased the yield more than two-fold by inserting three copies of Protegrin-1 in the GFP scaffold. The immunogold electron micrographs showed that the expressed Protegrin-1 in the GFP scaffold forms large and small size aggregates in the core region of the inclusion body and become entirely nonfunctional, therefore not influencing the proliferation of *E. coli*. Our novel method will be applicable for diverse bacteriotoxic peptides which can be exploited in biomedical and pharmaceutical researches.

Protein overexpression by using recombinant technologies is a very efficient and simple system that minimizes production costs. Numerous heterologous expression systems have been developed to express bacteriotoxic proteins in *Escherichia coli* and other hosts. These systems inactivate the function of the proteins through soluble or insoluble tag fusions (*e.g.* ketosteroid isomerase (KSI), N^pro^, amido-phosphoribosyl transferase (PurF), histone fold domain, maltose-binding protein, glutathione *S*-transferase, thioredoxin, SUMO, and intein)^1^,^2^,^3^,^4^,^5^,^6^,^7^,^8^. However, to our knowledge none of the expression systems had met the demands for the large-scale production of bacteriotoxic proteins^7^,^8^,^9^,^10^, most likely because of the inability of the fusion system to completely inhibit their intrinsic toxicity or prevent proteolytic degradation^7^,^8^.

Antimicrobial peptides (AMPs) are molecules secreted from diverse organisms, including mammals as a part of the innate immune system^11^,^12^. AMPs received substantial interest due to their strong antimicrobial activity against a broad range of pathogenic microorganisms, including conventional antibiotic-resistant strains^13^,^14^,^15^. In total, more than 5,000 AMPs have been discovered or synthesized up to date^16^, and the human genome contains 111 genes that encode three different antimicrobial peptide families, including defensins, histatins, and cathelicidines^17^,^18^. The importance of these molecules as immune modulators, anticancer drugs and in shaping gut and airway microbiota has been well described^11^,^12^,^13^,^14^,^15^. Indeed, some AMPs are currently being studied in clinical trials^15^.

Very few AMPs were successfully produced in heterologous expression systems^4^,^5^,^6^,^7^. In addition, conventional methods to produce AMPs from natural sources or chemical synthesis are less favorable due to high cost in the production and often with difficulties synthesizing peptides longer in size due to complex impurities with low yield^19^. The inability to efficiently produce AMPs significantly hampers research on important classes of proteins that combat disease. What is more, the ability to produce functional AMPs would allow us to further easily study their properties and may even provide clinical applications in the future.

In this study, we developed a high efficiency expression system for producing bacterial toxic proteins, including AMPs, by using engineered green fluorescent protein (GFP) as a scaffold^20^, which is one of the most commonly used proteins in biological studies. To demonstrate the effectiveness of the expression system, we produced several peptides or proteins that have potent antimicrobial activities, including Protegrin-1 (PG-1)^21^,^22^, PMAP-36^23^, Buforin-2^24^, and the scorpion venom component Bactridin-1^25^. Additionally, we compared our method of AMP production to the two most commonly used methods. From the results, we show that our novel method is superior to the existing methods in producing AMPs and may be a useful tool in a variety of academic and pharmaceutical settings.

## Results

### Expression of recombinant PG-1 using conventional expression systems

In this study, we aimed to exploit various expression systems to develop a highly effective expression system for the production of active bacterial toxic proteins. We used PG-1, one of the most difficult AMPs to be expressed in bacterial expression systems, as a reference to evaluate the efficiencies of the different methods^26^. We cloned PG-1 into pET30b, a commonly used bacterial expression vector. However, we were unable to obtain any transformants, suggesting that leaky expression of PG-1 from the plasmid causes toxicity to the host. To overcome this, we co-transformed cells with the pLysS plasmid, which produces an inhibitor of T7 RNA polymerase, to abolish the leaky expression and were able to obtain transformants harboring the PG-1 expression vector. However, we observed toxicity and an absence of bacterial growth when PG-1 expression was triggered in the cells (data not shown).

As an alternative strategy, we attempted to express PG-1 in the cytoplasm of *Pichia pastoris* GS115 yeast using both buffered methanol and minimal methanol medium. Protein samples were collected at different time intervals during the induction and were subjected to SDS-PAGE (Supplementary Fig. 1a,b). However, we did not detect PG-1 expression perhaps due to PG-1 levels being below a detectable limit using this procedure or proteolytic cleavage of PG-1 by intracellular proteases. Taken together, these results indicate that conventional bacterial or fungal expression systems cannot effectively produce bactericidal proteins.

### Expression of AMPs using a traditional fusion partner system

**Figure 1:**
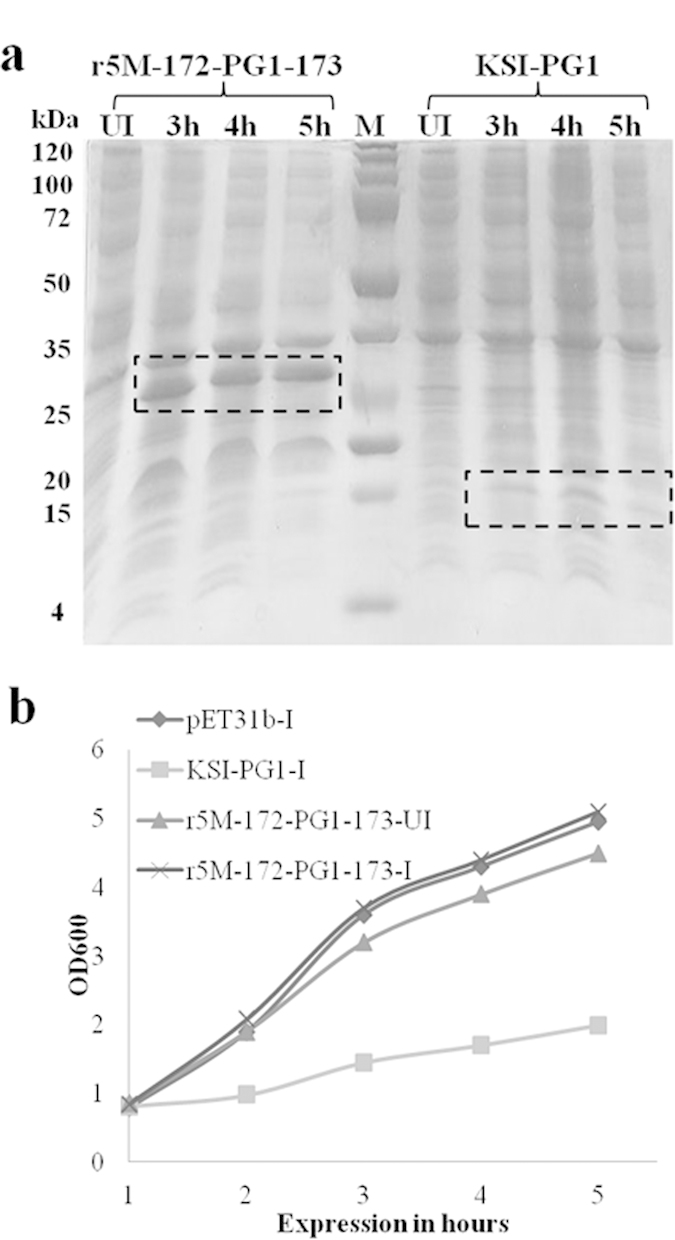
Comparison of protein expression levels and bacterial growth of *E. coli* r5M-172-PG1-173 and KSI-PG1 fusion systems. (**a**) Comparison of protein production efficiency of r5M-172-PG1-173 in pET30b and KSI-PG1 in pET31b. Total cellular protein samples were analyzed by 12% SDS-PAGE at different expression times. Lane UI: uninduced protein sample, lanes 1–3 were induced protein samples collected at 3, 4, and 5 h of expression, respectively, and M is molecular weight marker. The boxes indicate the expected size of r5M-172-PG1-173 (31 kDa) and KSI-PG1 (18.4 kDa). Results showed >7 fold increase in 172PG-1173 production compared to KSI-PG1. (**b**) Growth of *E.coli* carrying pET31b with KSI and PG-1 fused to KSI (KSI-PG1) after induction and r5M-172-PG1-173 in pET30b with and without induction with IPTG. The OD_600_ was measured for all of the constructs with or without induction every hour up to 5 h (I = induced and UI = uninduced).

Next, we tested whether fusing PG-1 to a commonly used tag (KSI) would increase the expression of PG-1. The ketosteroid isomerase tag is highly insoluble in nature and proteins fused to KSI are trapped in inclusion bodies^8^. PG-1 fused to KSI in pET31b (KSI-PG1) was expressed in *E. coli*. Bacterial growth was measured at OD_600_, and total proteins were collected at different time points during the induction of KSI-PG1. SDS-PAGE analysis revealed the expression of KSI-PG1 at the expected size (Fig. 1a). However, we found a significant decrease in the growth rate of cultures expressing KSI-PG1 compared to untagged KSI protein (Fig. 1b). These data indicate that a well-known fusion partner system also possesses limitations for the efficient production of toxic proteins.

### Construction of a GFP-scaffold vector system for the expression of bacterial toxic proteins

**Figure 2:**
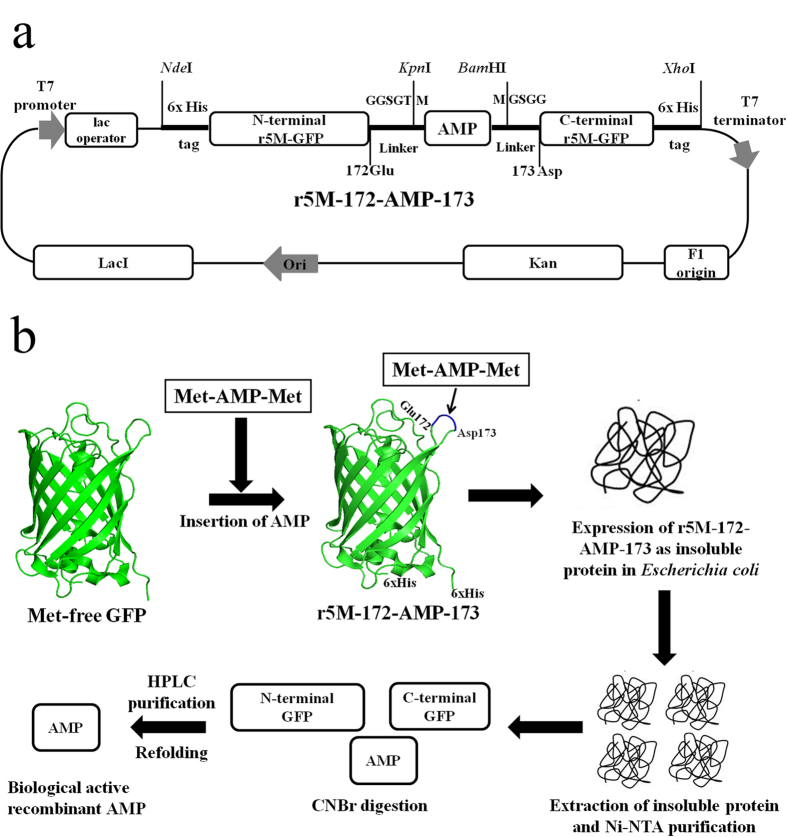
The vector map of the r5M-172-AMP-173 expression construct and the production strategy of antimicrobial peptides using r5M-GFP system in *E. coli.* (**a**) AMPs were inserted between 172 and 173 amino acids positions of r5M-GFP. Loop structure forming linker sequences, GGSGT and GSGG, are used for cloning and indicated in amino acids. The letter “M” flanking both 5′ and 3′ side of AMP indicates methionine. The restriction sites and 6x His-tag at both N- and C-terminals are indicated. (**b**) AMPs are cloned into a loop region of methionine-free GFP (r5M-172-AMP-173) and expressed as insoluble proteins in *E. coli*. The insoluble proteins are extracted and purified by Ni-NTA and cleaved at methionine sites using cyanogen bromide. Finally, AMPs are purified by high performance liquid chromatography.

We used GFP as a scaffold to insert the toxic proteins in the amino acid position 172 of the loop region of GFP^27^. DNA sequences encoding PG-1 and PMAP-36 were prepared by PCR amplification using pig cDNA and Buforin-2 and Bactridin-1 were chemically synthesized and flanked with methionine (Met) codons, respectively. The AMP sequences were inserted into a highly expressible GFP, which is devoid of internal Met residues^20^,^28^ to produce a DNA fragment called r5M-172-AMP-173 (Fig. 2a). The flanked Met residues at the beginning and end of the target AMPs allow chemical cleavage by cyanogen bromide (CNBr), releasing the AMPs for further purification (Fig. 2b). We prepared five different GFP-scaffold expression constructs from the four AMP genes by inserting the corresponding AMP monomer or trimer sequences within the GFP gene, followed by subcloning into the pET30b vector backbone (providing a His-tag for purification). The resulting constructs containing *PG-1*, (*PG-1*)_3_, *PMAP-36*, *buforin-2* and *bactridin-1* were successfully transformed and expressed in *E. coli*.

### Production of recombinant AMPs using the GFP-scaffold vector system

After optimization of culture conditions (see Methods), the transformants were cultured and harvested after 5 h of induction. The amount of total recombinant protein was approximately 25% of the total cellular proteins, indicating high efficiency in the expression of the recombinant proteins. From 1 liter of bacterial culture in Luria broth medium (LB), a total of approximately 1.3–1.4 g of insoluble proteins were measured by Bradford assay (Table 1) and subsequent SDS-PAGE analysis showed that 80–90% of the total insoluble pellet was recombinant target proteins (Supplementary Fig. 2).

**Figure 3:**
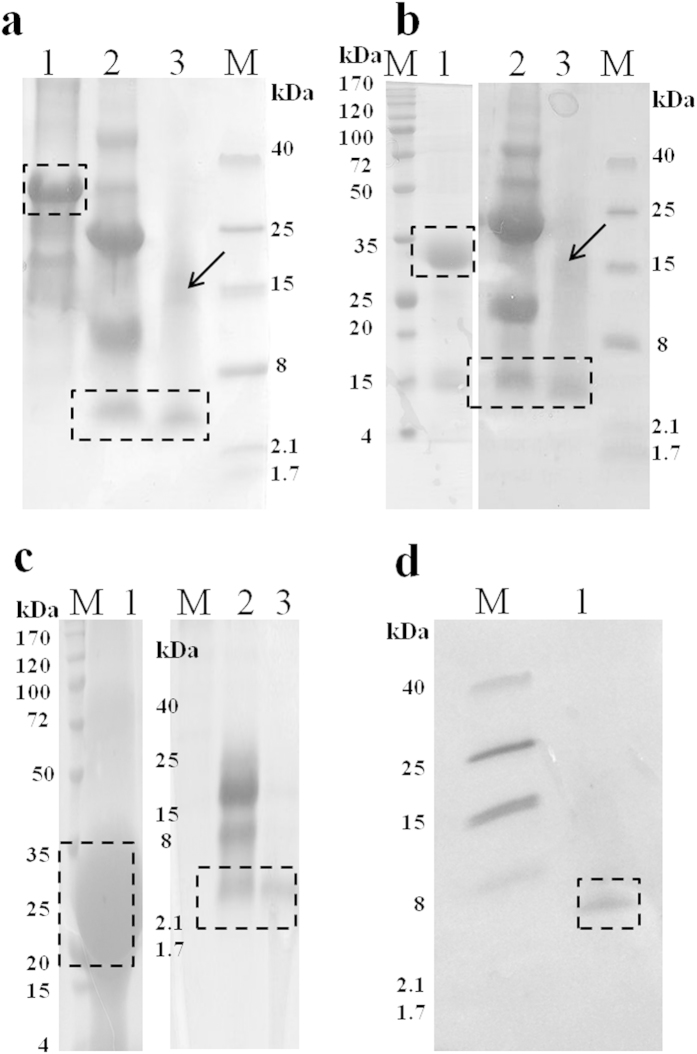
Purification of PG-1, PMAP-36, and Buforin-2 and confirmation of PG-1 expression. Insoluble protein purification by affinity chromatography, CNBr digestion of affinity-purified target proteins, and reverse-phase high performance liquid chromatography (RP-HPLC) of CNBr-digested r5M-172-PG1-173 (**a**) r5M-172-PMAP36-173 (**b**) and r5M-172-Bf2-173 (**c**) respectively. Lane 1: affinity purification of insoluble protein; lane 2: CNBr digestion of purified insoluble protein; lane 3: final RP-HPLC purification of PG-1 (**a**) PMAP-36 (**b**) and Buforin-2 (**c**) respectively, and lane M is molecular weight marker. The boxes indicate the expected size of the target protein after the various purification steps for PG-1 (2.4 kDa), PMAP-36 (4.2 kDa), and Buforin-2 (2.3 kDa). In lane 3, the purified peptides with higher molecular weights (indicated by an arrow) show the occurrence of AMP multimerization. The samples were loaded onto either 16% Tris-Tricine or 12% SDS-PAGE gels. (**d**) Confirmation of the presence of purified PG-1 using a rabbit anti-PG-1 antibody.

The His-tagged insoluble proteins were purified further using Ni-NTA chromatography and the purified target proteins were pooled with purity determined by SDS-PAGE. Subsequently, the proteins were dialyzed, lyophilized and quantified. Although, the recovery of the target protein after different steps of purification resulted in around 20 to 30% yield, still it was a sufficient amount for further process. The purification yield of the target protein could be improved by processing the lysis buffer after different washing steps described in Methods. The purified target protein was treated with CNBr in the presence of formic acids. The subsequent RP-HPLC purification of the target proteins PG-1 (18aa, 2.4 kDa), PMAP-36 (36aa, 4.2 kDa), and Buforin-2 (21aa, 2.3 kDa) yielded 12-14 mg of the purified AMPs (Table 1) and the purity was >95% (Fig. 3a–c). In order to validate the successful purification of PG-1, the result was evaluated by western analysis using an anti-PG-1 antibody^29^, revealing a band corresponding to PG-1 at 2.4 kDa (Fig. 3d).

The expression level of PG-1 using the GFP scaffold increased 7-fold or greater compared to KSI-PG1 by densitometric analysis of the target band in SDS-PAGE while comparing total cellular proteins (Fig. 1a), and we obtained similar results for other AMPs (Table 1). Additionally, we also expressed Bactridin-1 (61aa, 7.1 kDa) using the GFP scaffold system (Supplementary Fig. 3) as a proof-of-principle to show that the GFP scaffold system can support the production of bacteriotoxic proteins that have greater molecular masses although we only confirm its expression using SDS-PAGE without further purification. These results demonstrate the high efficiency production of bacterial toxic proteins using the r5M-GFP system over other available methods.

### Enhancement of target protein expression using multiple copies of PG-1 in r5M-GFP

The expression level of target proteins could potentially be enhanced by either favoring fermentation conditions to the host like continuous fermentation in nutrient-rich media or increasing the copy number of genes encoding the target proteins (Supplementary Table 2)^30^,^31^,^32^,^33^,^34^,^35^,^36^,^37^. Therefore, we developed a construct with three copies of PG-1 and evaluated yield improvement. Our results show that the r5M-GFP construct with three copies of PG-1 (r5M-172-(PG-1)_3_-173) increased the total yield by 2.3-fold compared to the single copy construct (Table 1), suggesting that this method could meet the requirement of pharmaceutical production at low cost.

### Biological activity of the recombinant AMPs

To confirm the antimicrobial activity of the recombinant AMPs produced using the methods developed in this study, purified recombinant proteins, including PG-1, PMAP-36, and Buforin-2, were used in minimum inhibitory concentration (MIC) assays with *E. coli* ATCC 25922, *Pseudomonas aeruginosa* ATCC 27853, and *Staphylococcus aureus* ATCC 29213. Although there were differences in MIC values against different microorganisms depending on the characteristics of each AMP, each recombinant AMP showed broad-spectrum activity similar to their reported MIC values^22^,^23^,^24^ (Table 2). These data reveal that the recombinant AMPs produced using our method has similar biological activity compared to chemically synthesized AMPS.

### Immunogold transmission electron microscopy of expressed r5M-172-PG1-173 in *E. coli*

**Figure 4:**
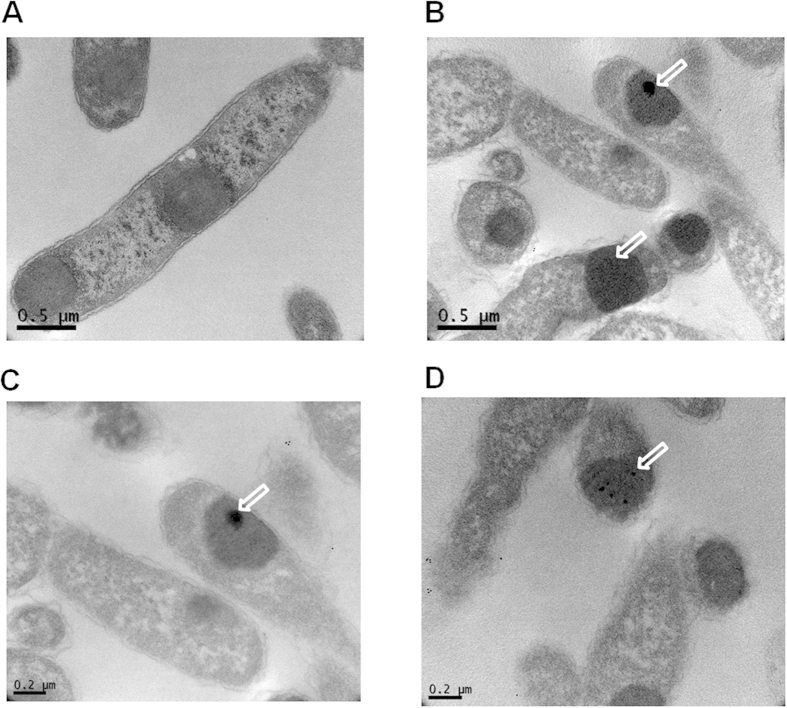
Immunogold transmission electron micrographs of expressed r5M-172-PG1-173 inclusion bodies in *E. coli* using PG-1 specific antibodies. (**A**) Expression of r5M-172-PG1-173 as insoluble inclusion bodies was clearly visible inside the *E. coli* cytoplasm. (**B–D**) The expressed inclusion bodies of r5M-172-PG1-173 were immunogold labeled using rabbit-anti PG-1 antibody. The expressed PG-1 was shown as dark spots within the inclusion bodies and indicated by arrows.

To address the reason for the expression of r5M-172-PG1-173 not being apparently toxic to *E. coli*, immunogold transmission electron microscopy was employed to localize the produced peptide using PG-1 specific antibody. The electron micrograph clearly showed that the r5M-172-PG1-173 are present as insoluble aggregates in the *E. coli* cytoplasm (Fig. 4A). Interestingly, the PG-1 produced in the *E. coli* formed a single large aggregate together with several smaller aggregates buried inside the inclusion bodies. Although the penetration of the antibody into the inclusion bodies is variable^38^,^39^, we were able to see that most of the PG-1 specific signals were identified inside of the inclusion bodies. These results suggest that the expressed PG-1 in the r5M-GFP scaffold was buried within the inclusion body and consequently prevent PG-1 from being exposed or interacting with the inner cell wall which is likely to cause cell damage.

## Discussion

Preparing sufficient amounts of target molecules can generate an experimental bottleneck if the ability to produce the molecule is hindered by experimental pitfalls. AMPs are prime candidates to serve as broad-spectrum antimicrobials and are molecules that help to shape the microbiota in the gut and airways, playing critical roles in animal health^40^,^41^. However, our understanding of these molecules is limited because of technical difficulties in producing them in sufficient quantities with reasonable costs and simplistic methods. Bacterial expression systems often produce recombinant proteins with high efficiency and are preferred methods to generate a variety of proteins^7^,^8^,^9^,^10^. Here, we developed a method to efficiently produce highly potent antimicrobial peptides using a GFP scaffold and demonstrated its incomparable efficiency and versatility over existing methods.

It has been shown that the GFP backbone is rigid and tolerant to short peptide insertions into its loops^42^,^43^. Kobayashi *et al.* exploited the loop region of GFP for the successful insertion of a novel 67 amino acid long multifunctional tag at 172 Asp position without compromising the activity of GFP and proved to be easily accessible by relevant antibodies and applicable to affinity tag purification^27^. Therefore, we chose this position within the GFP scaffold to insert toxic proteins to search for a potentially more productive way to produce AMPs than the use of previously reported fusion protein methods.

A previous study showed that r5M-GFP behaves as a soluble protein in aqueous solution^20^. Thus, we were surprised to find that AMPs fused with the GFP scaffold were completely insoluble. This was probably due to the significant number of positively charged residues of AMPs and likely played a role in completely abolishing the toxic effect and proteolytic degradation of the peptides inserted into the GFP scaffold. Therefore, the level of AMP expression in our system was much greater than any other previously reported method. It has been reported that the expression of engineered insoluble GFP in bacteria does not display any apparent toxicity to host cells^20^,^28^. Moreover, unfolded proteins in the cytoplasm that do not result in pathological aggregates have intrinsically large net charges and become insoluble^44^,^45^. Indeed, the immunogold transmission electron micrographs showed that the PG-1 clustered primarily inside of the inclusion bodies as aggregates (Fig. 4). The growth rate of r5M-172-PG1-173 with or without induction had no effect on growth of *E. coli*, indicating that the accumulation of insoluble aggregates in the cytoplasm does not affect the proliferation of *E.coli* (Fig. 1b and Supplementary Table 1), which is consistent to the result of the electron microscopy. Constant production yields of several AMPs in this study suggest that they all form structurally similar inclusion bodies like PG-1 irrespective of expressed AMPs.

It has been shown that some AMPs were successfully expressed as soluble fusion proteins by blocking either N- or C-termini of the toxic peptide^4^,^5^,^6^. However, incomplete suppression of the toxic effects of the proteins or partial proteolytic degradation of the soluble fusion proteins prevented the general use of that system for the expression of toxic proteins. Toxic proteins fused to insoluble tags may produce either neutral or unfavorable aggregates depending on the nature of proteins and quite often interfere with host metabolism or growth rate^46^.

In order to investigate the effect of r5M-172-PG1-173 expression on bacterial growth, the proliferation of bacteria was compared under induced and un-induced conditions (Supplementary Table 1). The results showed no difference in the growth rate between the two conditions, indicating that the insolubility of r5M-172-AMP-173 did not interfere with the growth rate of *E. coli*. The KSI tag was used to produce low expression levels of AMPs, including lactophoricin, dermcidin, and PFR (Supplementary Table 2)^8^,^30^. To evaluate the efficiency of our GFP scaffold system, we compared the efficiency of protein production using the KSI tag and our GFP-based system. In contrast to the GFP scaffold system, the expression of KSI-PG1 was detrimental to bacterial growth (Fig. 1b and Supplementary Table 1). The accumulation of structurally unfavorable KSI-PG1 inclusion aggregates might alter *E. coli* metabolism or growth rate^46^. Alternatively, the KSI-PG1 fusion system may be unable to completely abolish the activity of PG-1.

Although the use of continuously supplied nutrient rich media can increase protein expression as shown in previous studies (Supplementary Table 2), its benefit comes with high cost and requires a special fermentation system which is not easily accessible for many laboratories. Our data revealed an extremely high efficiency of AMP production by using multiple copies of the AMP gene in the r5M-GFP system with LB medium under standard laboratory conditions. This demonstrates that the GFP scaffold system may be an ideal method for a variety of applications in many laboratories. Furthermore, we confirmed the successful expression of three highly potent AMPs ranging in molecular masses from 2.3 to7.1 kDa.

Our system has advantages that meet several industrial standpoints, including robust expression, easy collection of inclusion bodies, prevention of proteolytic degradation, and complete abolishment of the intrinsic toxicity induced by the target proteins. The major drawbacks of our system are the inapplicability of our method for proteins that have Met residues in their coding sequence (not including the start codon that encodes Met) because of the purification step involving CNBr cleavage and its toxic nature. Although AMPs with Met residues in the coding region seem to be rare, this difficulty could be overcome by a genetic manipulation which replaces Met for the CNBr cleavage with other amino acids such as Asp-Pro, which would allow for the release of AMPs from GFP scaffold by less toxic and inexpensive acid cleavage^30^. As another limitation, the r5M-GFP system is not suitable for the production of cyclic AMPs which require a separate cyclization process of produced peptides or proteins.

In summary, we demonstrate the successful development of a method for the large-scale production of biologically active antimicrobial peptides and proteins which can achieve high efficiency production of long and linear peptides with complex disulfide bonds. Our method will likely be useful for producing sufficient amounts of bacterial toxic proteins, including AMPs, for a variety of applications.

## Methods

### Construction of r5M-172-AMP-173 in pET30b

*r5M-GFP* devoid of internal methionine (Met) was cloned into *Nde*I and *Xho*I (New England BioLabs (NEB), Tokyo, Japan) sites in pET30b (Novagen, Germany) vector. This vector was used to insert the AMP sequences using overlap extension polymerase chain reaction (OE-PCR)^20^ with the primers described in Supplementary Table 3. First, AMPs such as *PG-1* and *PMAP-36* were PCR amplified with pfu-x enzyme (Solgent, Seoul, Korea) using pig cDNA with the following sets of primers: KpnI-M-PG-1-For and 172linker-M-PG-1-R, and 172linker-M-PMAP-F and 172linker-M-PMAP-R, respectively (Supplementary Table 3). Cycling condition to amplify *PG-1* and *PMAP-36* were 95 °C for 3 min followed by 35 cycles of 95 °C for 1 min, 56 °C for 30 sec, and 72 °C for 45 sec, with a final extension of 72 °C for 10 min. The primers used to amplify AMPs have linkers (the detailed linker sequences were shown in Supplementary Table 3) and Met sequences for subcloning and purification, respectively. Secondly, *r5M-GFP* in pET30b was used as the template to generate two fragments, N-terminal of *r5M-GFP* to amino acid position 172 (Glu) and amino acid position 173 (Asp) to the C-terminal using primer sets, r5M-NdeI-6xHis-For and 172ATG-Rev1, and 172ATG-For1 and r5M-XhoI-Rev, respectively (Supplementary Table 3). These two DNA fragments along with amplified fragments of *PG-1* and *PMAP-36* were gel-purified using 1.5% agarose (Qiagen Gel Extraction Kit, Netherland). Finally, equimolar quantities of N- and C-terminal fragments of *r5M-GFP* along with *PG-1* and *PMAP-36*, respectively, were assembled together by overlapping linker sequences present at each fragment. Subsequently, overlap PCR was carried out using primers, r5M-NdeI-6xHis-For and r5M-XhoI-Rev. The resultant DNA fragments (r5M-172-AMP-173) containing either *PG-1* or *PMAP-36* were inserted in the loop region of *r5M-GFP* between amino acid positions 172 and 173 with linker sequences followed by Met flanking the AMP sequences at both termini (r5M-172-AMP-173) which was necessary for cyanogen bromide (CNBr) (Sigma Aldrich, MO, USA) cleavage. This fragment was cloned into pET30b harboring a his-tag at both termini in the *Nde*I and *Xho*I sites (Fig. 2a).

Similarly, r5M-172-(PG1)_3_-173 was prepared by annealing full-length *PG-1* DNA fragment primers, PG-1-ATG-For and PG-1-TAC-Rev, with their overhang sequences of TAC and ATG sequences in the 5′ end of forward and the 3′ end of reverse primers, respectively (Supplementary Table 3). The annealed double stranded *PG-1* fragments were multimerized by ligating the PG-1 monomers by T4 DNA Ligase (NEB). The ligated products were run on a 3% low melting agarose gel and DNA bands appearing above 150 bp and within 300 bp were excised from the gel and purified using phenol-chloroform. The purified DNA fragment was cloned into *Alw*NI (NEB) digested r5M-GFP and pET30b plasmids, respectively, which was prepared by insertion of a *Alw*NI site at position 172 using site-directed mutagenic primers (172AlwNI-ATG-For and 172AlwNI-ATG-Rev) (Supplementary Table 3) according to the manufacturer’s protocol (Stratagene, CA, USA) to add three copies of the PG-1 insert at the position 172 of r5M-GFP. Additionally, *bactridin-1* and *buforin-2* were chemically synthesized (GenScript USA Inc., NJ, USA), and these DNA fragments were also inserted into position 172 of r5M-GFP and cloned into pET30b and called r5M-172-Bf2-173 and r5M-172-Bact1-173, respectively.

### Preparation and expression of PG-1 using KSI fusion and a yeast system

The *PG-1* DNA fragments, PG-1-ATG-For and PG-1-TAC-Rev, (Table S3) were annealed and purified using phenol chloroform (Sigma Aldrich). The annealed DNA fragment contained ATG and TAG overhangs were ligated into the *Alw*NI digested pET31b vector (Novagen) downstream of the *KSI* gene (KSI-PG1) and transformed into *E. coli* BL21 (DE3) (Invitrogen, CA, USA). When the culture reached an OD_600_ of 0.6 in LB medium, the expression of the insert was induced with 0.1 mM IPTG (Sigma Aldrich). The pET31b construct carrying KSI was expressed in the same way. At various time intervals, cell viability was estimated and proteins were extracted and separated in SDS-PAGE.

*PG-1* was PCR amplified using the primer set (pPIC-BamHI-PG1-For and PG1-EcoRI-pPIC-Rev, Table S3) using pig cDNA. The amplified *PG-1* fragment and pPIC3.5 vector (Life Technologies, Seoul, South Korea) were digested with *Bam*HI and *Eco*RI (NEB), ligated, and transformed. The plasmid pPIC3.5 containing *PG-1* was linearized using *Sac*I (NEB) and integrated into the *P. pastoris* GS115 (Life Technologies) genome using an electroporator (MicroPulser^TM^, Bio-rad, CA, USA). Protein expression was carried out using the *Pichia* Expression Kit (Life Technologies) in buffered and minimal methanol medium according to the manufacturer’s protocol.

### Expression and insoluble protein extraction of r5M-172-AMP-173

The constructs r5M-172-PG1-173, r5M-172-PMAP36-173, r5M-172-Bf2-173, r5M-172-(PG1)_3_-173 and r5M-172-Bact1-173 in pET30b were transformed into *E. coli* BL21. All of the constructs were grown in 1 L Luria broth (LB) at 37 °C and the cultures were induced with 0.1 mM IPTG when the OD_600_ of the culture reached 0.6 to 0.8. The proteins were induced for 5 h and the cells were harvested by centrifugation at 8,000 rpm for 10 min at 4 °C. Insoluble extractions were carried out by disrupting the cells by sonication in lysis buffer (20 mM sodium phosphate buffer pH 7.4 containing 100 mM sodium chloride, 0.1 mM PMSF, 1 mM DTT) (Sigma Aldrich). The lysate was spun at 13,000 rpm for 20 min and the soluble and insoluble fractions were collected and estimated by Bradford assay. In order to purify the target protein by removing cellular debris, nucleic acids and cytosolic proteins from insoluble fraction as a pre-column process, the insoluble fraction pellet was washed by resuspending it in the lysis buffer (40 ml per 1 L culture pellet), and then DNase (0.01 mg/ml), lysozyme (0.1 mg/ml), and 0.5% Triton-X 100 (Sigma Aldrich) were added to the insoluble fractions and incubated at room temperature for 20 min. The samples were centrifuged at 8,000 rpm for 10 min at 4 °C and the insoluble fractions pellet was collected. Next, the insoluble fraction pellet washes were repeated two or three times without the addition of DNase and lysozyme. Finally, the insoluble fraction pellets were resuspended in 20 mM sodium phosphate buffer pH 7.4 containing 8 M urea and 30 mM imidazole. The insoluble fraction proteins were purified by Ni-NTA column chromatography (GE Healthcare Bio-Sciences, Sweden) by using a standard denaturation purification protocol. Eluted fractions were analyzed for purity by SDS-PAGE and dialyzed against deionized water at room temperature.

### RP-HPLC purification and refolding

The insoluble precipitates of r5M-172-AMP-173 were lyophilized, quantified by Bradford assay and dissolved in 70% formic acid. CNBr was added to this mixture (100 mg/ml) and incubated in the dark for 24 h to cleave N- and C-terminal residues of GFP flanking the AMP. Formic acid and CNBr were removed by lyophilization and the purity of the samples was checked at each stage by 16% Tris-Tricine PAGE. Both PG-1 and PMAP-36 were purified using preparative reverse-phase HPLC (Deltapak C18 column 7.8, 300 mm, Water, Tokyo, Japan) in a linear gradient of acetonitrile 5 to 90% in 60 min in 0.1% trifluoroacetic acid at a flow rate 2.5 ml/min. Absorbance was monitored at both 220 nm and 280 nm and corresponding peaks were checked using 16% Tris-Tricine PAGE. Purified fractions were lyophilized and suspended in 20 mM sodium phosphate buffer pH 7.4 containing 8 M urea, 5 mM reduced glutathione and 0.5 mM oxidized glutathione. The mixture was dialyzed against deionized water and purified peptides were lyophilized.

### Antimicrobial activity assay

Microbial viability assay kit-WST^®^ (Dojindo Molecular Technologies, Japan) was used to analyze antimicrobial activity according to the manufacturer’s protocol. *E. coli* (ATCC 25922), *S. aureus (*ATCC 29213), and *P. aeruginosa* (ATCC 27853) used for MIC assays were obtained from American Type Culture Collection (VA, USA). We used 10^4^ cfu/ml of bacteria in Mueller-Hinton broth, added this to various concentrations of purified recombinant AMPs, and incubated at 37 °C for 6 h in a 96 well plate. Coloring reagents were added and incubated for 2 h at 37 °C. Finally, the plates were read at 405 nm using a Bio-rad Microplate reader (Bio-rad). Minimal inhibitory concentration (MIC) of AMPs is defined as the lowest concentration of AMPs that shows 100% growth inhibition.

### Western blot analysis

Purified recombinant PG-1 (~3 μg) was suspended in 1× SDS loading dye (ThermoScientific, USA) and loaded onto a 16% Tris-Tricine SDS-PAGE gel along with low molecular weight markers (ThermoScientific, USA). The peptides were separated and electro-transferred onto Hybond-P PVDF membrane (GE Healthcare Bio-Sciences, Sweden) and blocked with 5% skim milk containing 0.5% Triton-X 100 in 1× PBS and incubated overnight at 4°C with rabbit anti-PG-1 antibody (1:1000) which was previously developed^29^. The membrane was incubated with anti-rabbit IgG-HRP (1:2000; Santa Cruz, CA, USA) for 2 h at room temperature. The blot was visualized using TMB liquid substrate (Sigma Aldrich, St. Louis, MO, USA).

### Immunogold transmission electron microscopy

*E. coli* BL21 containing r5M-172-PG1-173 was expressed for three hours by IPTG induction and the cells were harvested by centrifugation. Cells pellet were washed twice with 1X PBS and 0.4 OD_600_ cells was fixed with 2% paraformaldehye containing 2.5% glutaraldehyde (Sigma Aldrich) in PBS for an hour at 4°C. The cells were washed five times with PBS and used for immunogold labeling. To improve reagent penetration, cells were treated with 0.5% Triton-X 100 for 15 minutes at room temperature and washed three times with 1X PBS. Subsequently, cells were treated again with 0.01N HCl for 10 min and washed similarly with PBS. One batch of cells were used for immunogold labeling and another batch without labeling. Blocking was carried out with 5% bovine serum albumin (BSA) containing goat serum in PBS for 1 hour at 4 °C. Cells were incubated with rabbit anti-PG-1 antibodies (1:500) diluted in PBS containing 0.5% BSA for overnight at 4 °C. The cells were washed for 5 times with PBS for 10 min each. Goat anti-rabbit IgG secondary antibodies with 10 nM gold conjugated (Sigma Aldrich) was diluted in PBS containing 0.5% BSA and incubated similar to primary antibody. Excess antibodies were washed for 5 times with PBS. Both labeled and non-labeled cells were post-fixed in 2.5% glutaraldehyde in PBS for 2 hours at 4 °C and washed twice with PBS for 10 min. Cells were treated with 0.5% Osmium tetraoxide (Sigma Aldrich) in PBS for 15 min at 4 °C. Subsequently, the bacterial pellets were dehydrated through series of graded acetone (50%, 70%, 90%, and 100%) for 10 min each and finally rinsed in propylene oxide three times with 10 min each. The cells were embedded into Spur resin according to manufacture protocol (SPI supplies, PA, USA). Ultrathin sections were obtained using ultramicrotome and stained with 4% uranyl acetate and lead citrate. Sections were observed under TEM (Hitachi H7650, Japan).

## Additional Information

**How to cite this article**: Soundrarajan, N. *et al.* Green fluorescent protein as a scaffold for high efficiency production of functional bacteriotoxic proteins in *Escherichia coli*. *Sci. Rep.* **6**, 20661; doi: 10.1038/srep20661 (2016).

## Supplementary Material

Supplementary Information

## Figures and Tables

**Table 1:** Yield of purified recombinant AMPs at various steps of purification.

Purification steps	Yield (mg/L)			
	PG-1	PMAP-36	Buforin-2	(PG-1)_3_^a^
Total Insoluble protein^b^	~1400	~1300	~1320	~1350
Ni-NTA Purification^b^	~250	~220	~232	~241
RP-HPLC^c^	12 to 14	9 to 12	10 to 12	27 to 29

^a^Three copies of PG-1 was inserted in the scaffold of r5M-GFP.

^b^Determined by Bradford assay.

^c^Freeze-dried protein samples were measured by microbalance.

**Table 2:** Antimicrobial activities of the purified recombinant AMPs.

AMPs	Minimal inhibitory concentration (μg/ml)		
	*E. coli*	*P. aeruginosa*	*S. aures*
PG-1	7	15	10
PMAP-36	10	11	9
Buforin-2	6	9	8
