# Supplementary material for: Green fluorescent protein as a scaffold for high efficiency production of functional bacteriotoxic proteins in Escherichia coli
Source: Sci Rep. 2016 Feb 11;6:20661. doi: 10.1038/srep20661 (PMC4749965; doi:10.1038/srep20661)
Supplement: Supplementary Information [file srep20661-s1.doc]

**Green fluorescent protein as a scaffold for high efficiency production of functional bacteriotoxic proteins in *Escherichia coli***

Nagasundarapandian Soundrarajan^a^, Hyesun Cho^a^, Byeong Yong Ahn^a^, Minkyung Choi^a^, Le Minh Thong^a^, Hojun Choi^a^, Se-Yeoun Cha^b^, Jin-Hoi Kim^a^, Choi-Kyu Park^c^, Kunho Seo^d^ and Chankyu Park^1^*

**SUPPLEMENTARY FIGURES AND TABLES**

**Figure 1 (a) and (b)**


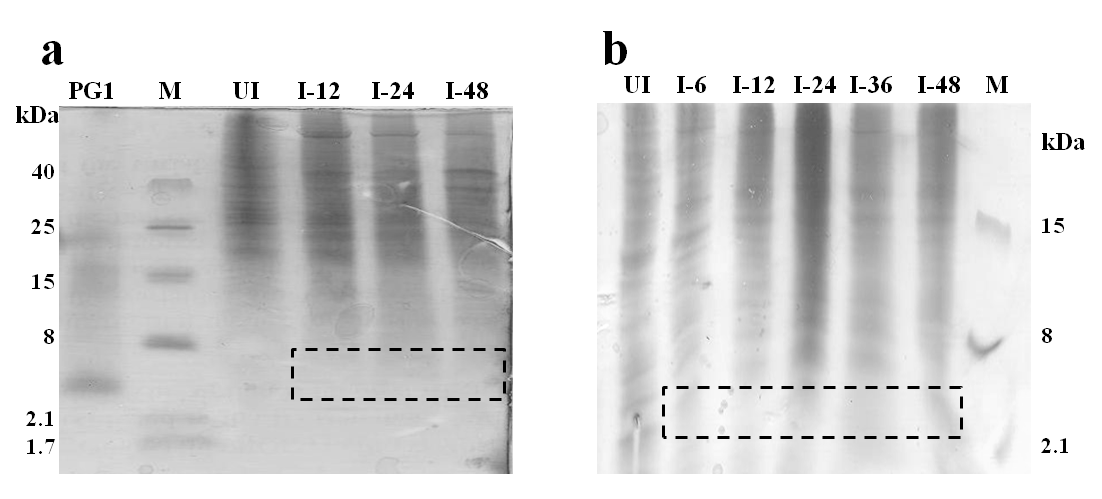


**Figure 1. Protein expression profile of PG-1 and the effect of inactivation of neutral proteases in *Pichia pastoris*.** (**a**) Total cellular protein of samples from *Pichia pastoris* GS115 containing PG-1. PG-1: purified PG-1 as marker; M: molecular weight marker; UI: uninduced protein sample; lane I-12, I-24, and 1-48 were induced protein samples collected at 12, 24, and 48 h, respectively. Expression of PG-1 was carried out in buffered methanol medium in *P. pastoris* GS115, but PG-1 expression was not detected by SDS-PAGE. Expression was carried out in buffered methanol medium and induced with 0.5% methanol, and induction was maintained by the addition of methanol every 24 h. (**b**) Total cellular proteins of samples from *P. pastoris* GS115 containing PG-1 expressed under acidic condition using minimal methanol medium to inactivate neutral proteases. Lanes I-6 to 1-48 was induced protein samples collected at 6, 12, 24, 36, and 48 h, respectively. The boxes indicate the expected size of PG-1 (2.4 kDa), but the expression of PG-1 is clearly not detectable. The cultures were induced with 0.5% methanol and induction was maintained by the addition of methanol every 24h.

**Figure 2 (a), (b), and (c)**

**
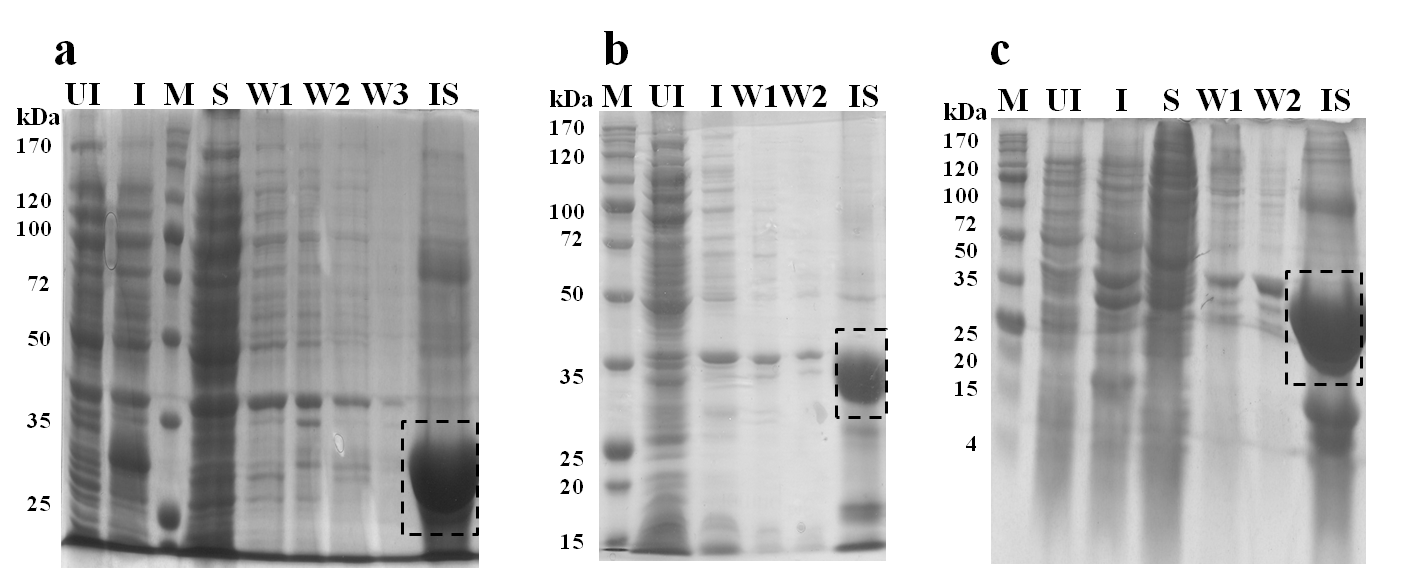
**

**Figure 2. Productivity of r5M-172-PG1-173, r5M-172-PMAP36-173, and r5M-173-Bf2-173 in pET30b expressed in *E. coli*.** Insoluble proteins were expressed for 5 h and extractions of r5M-172-PG1-173 (**a**), r5M-172-PMAP36-173 (**b**)*,* and r5M-172-bf2-173 (**c**) were carried out. Lane UI: uninduced protein sample; I: induced total cell protein after 5 h of expression; W1: first, W2: second, and W3: third insoluble extraction washes; IS: final insoluble extracted proteins. The boxes indicate the expected size of r5M-172-PG1-173 (31 kDa), r5M-172-PMAP36-173 (33.1 kDa), and r5M-172-bf2-173 (30.8 kDa), respectively, showed in 12% SDS-PAGE. The cultures were induced with 0.1 mM IPTG and expressed for 5 h at 37°C.

**Figure 3.**


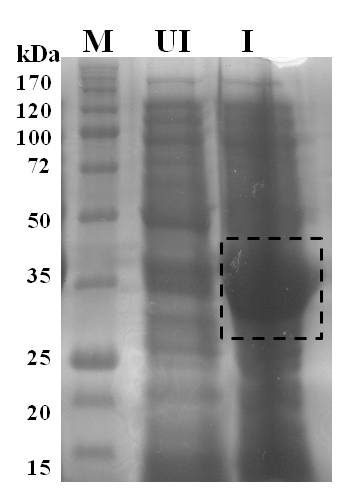


**Figure 3. Expression of r5M-172Bact1-173 in pET30b in *E. coli*.** Insoluble protein expression of r5M-172-Bact1-173. Lane UI: uninduced total cell protein sample; I: induced total cellular protein after 5 h of expression. The boxes indicate the expected size of r5M-172-Bact1-173 (35.6 kDa).

**Table 1: Viable cell counts of different expression constructs of PG-1 in *E. coli* BL21**

| **PG-1 expression constructs** |  | **Expression time (hours)** | | |  |
| --- | --- | --- | --- | --- | --- |
|  | **1.5** | | **2.5** | **3.5** | |
| **pET31b^a, 1^**  **KSI-PG1^b, 1^**  **r5M-172-PG1-173^2^**  **r5M-172-PG1-173^1^** | 2.7×10^7^  1.50×10^7^  3.7×10^8^  4.0 ×10^7^ | | 1.0×10^7^  9.0×10^6^  5.2×10^8^  2.6×10^8^ | 5.0×10^7^  1.82×10^6^  1.1×10^9^  5.0×10^8^ | |

^a^ pET31b carrying only KSI

^b^ pET31b carrying PG-1 fused to KSI

^1^Induced with 0.1 mM IPTG

^2^ Un-induced

**Table 2: Yield of recombinant AMPs produced using different methods**

| **Fusion partner** | **Target AMPs** | **Copy number^a^** | **Solubility^b^** | **Yield (mg/L)** | **Reference** |
| --- | --- | --- | --- | --- | --- |
| KSI^c^  GST^d^  6XHis-tag  Thioredoxin  Thioredoxin  MMIS^g^  MBP^h^  GST^i^  BCCP^j^ | PFWRIRIRR^e^  LF15-CA8  Hepcidin^e^  LL-37  LL-37^f^  Buforin-2^f^  ORBK  PR39  PG-1  PG-1  LL-37-inker- histatin-5 | 1  2  1  1  1  6  1  1  1  1  1 | Insoluble  Insoluble  Insoluble  Insoluble  Soluble  Insoluble  Soluble  Soluble  Insoluble | 10  10  6.8  2.6  40  107  3  1.9  1.1  0.55  0.41 | 30  31  32  33  34  35  36  26  37 |

^a^Number of copies of AMPs fused to the fusion partner protein to increase yield

^b^Target protein was produced as soluble or insoluble in cytoplasm of expression host

^c^Ketosteroid isomerase

^d^Glutathione *S*-transferase

^e^Different media composition such as high nutrient media to increase the growth and conditions used

^f^Different media composition and continuous fermentation was used with constant supply of nutrients

^g^Modified magainin intervening sequence

^h^Maltose-binding protein

^i^Both AMPs were fused together with GST

^j^Biotin carboxyl carrier protein was fused to both AMPs

**Table 3: PCR primers used in this study for the construction of expression vectors**

| **Primer names** | **Sequences (5’ to 3’)** |
| --- | --- |
| r5M-NdeI-6xHis-For | GATCATATGCATCACCATCATCACCATCAGAGCAAAGGCGAAG |
| r5M-XhoI-Rev | GATCTCGAGTTATTAATGGTGATGGTGATGGTG |
| 172ATG-For1 | ATGGGATCCGGTGGCGATGGCAGCGT |
| 172ATG-Rev1 | CATGGTACCAGA ACCACCTTCCACGTTATGAC |
| KpnI-M-PG-1-For | GCGGTTCTGGTGGTACCATGAGGGGAGGTCGCCTGTG |
| 172linker-M-PG-1-R | GCCACCGGATCCCATTCCTCGTCGACACAGACG |
| 172linker-M-PMAP-F | GGTTCTGGTACCATGGGACGATTTAGACGGTTG |
| 172linker-M-PMAP-R | CACCGGATCCCATCCCACAACCCA AGGGTA |
| PG-1-ATG-For | AGGGGAGGTCGCCTGTGCTATTGT AGGCGT AGGTTCTGCGTCTGT  GTCGGACGAGGA ATG |
| PG-1-TAC-Rev | TCCTCGTCCGACACAGACGCAGAACCTACGCCTACAATAGCACAG GCGACCTCCCCTCAT |
| 172AlwNI-ATG-For | GTACCCAGATGCTGGGATCCGGTGGCGATGGCAGCGT |
| 172AlwNI-ATG-Rev  pPIC-BamHI-PG1-For  PG1-EcoRI-pPIC-Rev | CAGCATCTGGGTACCAGA ACCACCTTCCACG  TTCGAAGGATCCACCATGGGTAGGGGAGGTCGCCTGTG  CCGCCCTAGGGAATTCTTATTATCCTCGTCCGAC |

Note: Linker sequences were underlined.
